# Comparison of Quantitative Computed Tomography and Dual X-Ray Absorptiometry: Osteoporosis Detection Rates in Diabetic Patients

**DOI:** 10.7759/cureus.23131

**Published:** 2022-03-13

**Authors:** Dheeraj Dheeraj, Udit Chauhan, Meenakshi Khapre, Ravi Kant

**Affiliations:** 1 General Medicine, All India Institute of Medical Sciences, Rishikesh, Rishikesh, IND; 2 Radiology/Interventional Radiology, All India Institute of Medical Sciences, Rishikesh, Rishikesh, IND; 3 Social Preventive Medicine, All India Institute of Medical Sciences, Rishikesh, Rishikesh, IND

**Keywords:** bone mineral density, quantitative computed tomography, dexa scan, osteoporosis, diabetes mellitus

## Abstract

Introduction

Diabetes mellitus (DM) adversely affects the skeletal system and is associated with an increased risk of osteoporosis and fragility fractures. This study aimed to assess the diagnostic accuracy of quantitative computed tomography (QCT) in osteoporosis detection in patients with DM.

Methods

A cross-sectional diagnostic accuracy study was conducted at the diabetic clinic of a tertiary care teaching hospital in North India. A total of 30 individuals with DM were subjected to spinal QCT and lumbar spine and hip dual x-ray absorptiometry (DXA). Sensitivity, specificity, positive predictive value (PPV), negative predictive value (NPV), and positive and negative likelihood ratios of QCT were measured against DXA and the diagnostic discordance between QCT and DXA was investigated.

Results

QCT, compared to the gold standard DXA, has a sensitivity/specificity of 92.8% (95% CI 92.4%-93.2%)/81.2% (95% CI 80.6%-81.8%). The PPV/NPV of QCT was 81.2% (95% CI 80.6%-81.8%)/92.8% (95% CI 92.4%-93.2%). The positive likelihood ratio/negative likelihood ratio was 4.95 (95% CI 4.79-5.11)/0.087 (95% CI 0.082-0.093). Area under the curve was 0.871 (95% CI 0.731-1.00). Minor diagnostic discordance was present in 36.6% of patients with diabetes.

Conclusion

The current study assessed the diagnostic accuracy of QCT in osteoporosis detection in people with diabetes. DXA is the gold standard diagnostic tool; however, its availability is limited. The current study showed that QCT is an excellent diagnostic tool. Based on these results, this study recommends that QCT may serve as a problem-solving investigation tool where DXA is unavailable, or it may be the primary investigation tool for bone mineral density measurement and osteoporosis detection if a dedicated DXA scanner is inaccessible. This study also recommends further investigating the feasibility of opportunistic osteoporosis screening in routine abdominal and chest CT. Finally, considering the silent nature of osteoporosis and the high prevalence of osteoporosis in individuals with diabetes, a proactive approach is required in the screening of osteoporosis.

## Introduction

Well-established late complications of diabetes mellitus (DM) are microvascular diseases, including nephropathy, retinopathy, and neuropathy, and macrovascular diseases such as acute coronary syndrome, peripheral vascular disease, and stroke. Chronic nonvascular complications mainly include genitourinary infections, gastroparesis, cataract, glaucoma, and periodontal disease. DM adversely affects the skeleton and is associated with an increased risk of osteoporosis and fragility fractures. Diabetes can also impair bone turnover and, therefore, skeletal integrity, and diabetic bone disease is often a neglected complication of diabetes.

Osteoporosis has no clinical manifestations until there is a fracture. Fractures result in significant morbidity and mortality. Moreover, osteoporosis results in a decreased quality of life, increased disability-adjusted life year, and a big financial burden. With an early diagnosis of this disease before fractures occur, by assessing the bone mineral density (BMD), and with early treatment, osteoporosis can be prevented. Considering these factors, screening for osteoporosis should be given a priority in individuals with diabetes. Technology promotion for assessing bone quality and quantity is required for early diagnosis and prompt management of osteoporosis in individuals with diabetes.

A meta-analysis by Vestergaard et al. showed that the hip fracture risk was increased in type 1 DM (T1DM; relative risk [RR] 6.94) and type 2 DM (T2DM; RR 1.38) patients compared to subjects without diabetes [1]. To better understand the association between DM and osteoporosis, Zeitoun et al. conducted a study on the femur of Zucker diabetic fatty and Zucker lean rats, showing that diabetic fatty rats exhibited significantly lower trabecular bone volume and number and higher trabecular separation than lean rats [2].

BMD measured by dual x-ray absorptiometry (DXA) is currently the gold standard for both osteoporosis diagnosis and the monitoring of treatment efficacy; however, its availability is limited. DXA is a projectional x-ray based method that has been proved to accurately and precisely quantify BMD at certain sites: the hip, lumbar spine, and distal forearm. To calculate the skeletal BMD, two x-ray beams with different energy levels are employed to deduct the patient's soft-tissue absorption. Both lumbar spine and hip scans expose one to about the same amount of radiation as a chest x-ray [3]. Quantitative computed tomography (QCT) can be performed on most commercially available CT scanners. QCT is a technique for measuring tissue density within a region of interest using a normal CT scanner. When compared to DXA, however, radiation exposure is significantly higher. The lumbar spine, hip, and tibia are among the QCT scanning locations. QCT produces a volumetric BMD, in opposition to the areal BMD of the DXA. QCT is also less susceptible to degenerative changes of the spine as compared with DXA.

Studies comparing QCT and DXA have found that QCT performs at par with DXA [4,5]. However, the diagnostic accuracy of QCT in osteoporosis detection in patients with diabetes remains unclear. Our study is aimed to assess the diagnostic accuracy of QCT in osteoporosis detection in patients with diabetes.

## Materials and methods

Patient selection

Inclusion criteria were age 50 years and above, diagnosed cases of DM as per American Diabetes Association (ADA) guidelines, having disease duration of more than one year, and having an indication of BMD measurement based on Fracture Risk Assessment Tool (FRAX) without BMD, i.e., a score of 8.4% or greater [6,7]. Participants having relative contraindication for bone densitometry, such as pregnancy, recent gastrointestinal contrast studies, and nuclear medicine tests, those suffering from any condition that prevents the patient from being properly positioned to get accurate BMD results, as well as those who were unable to get into the correct posture and/or remain immobile during the measurement were excluded [8].

Study design and sample size

**Figure 1:**
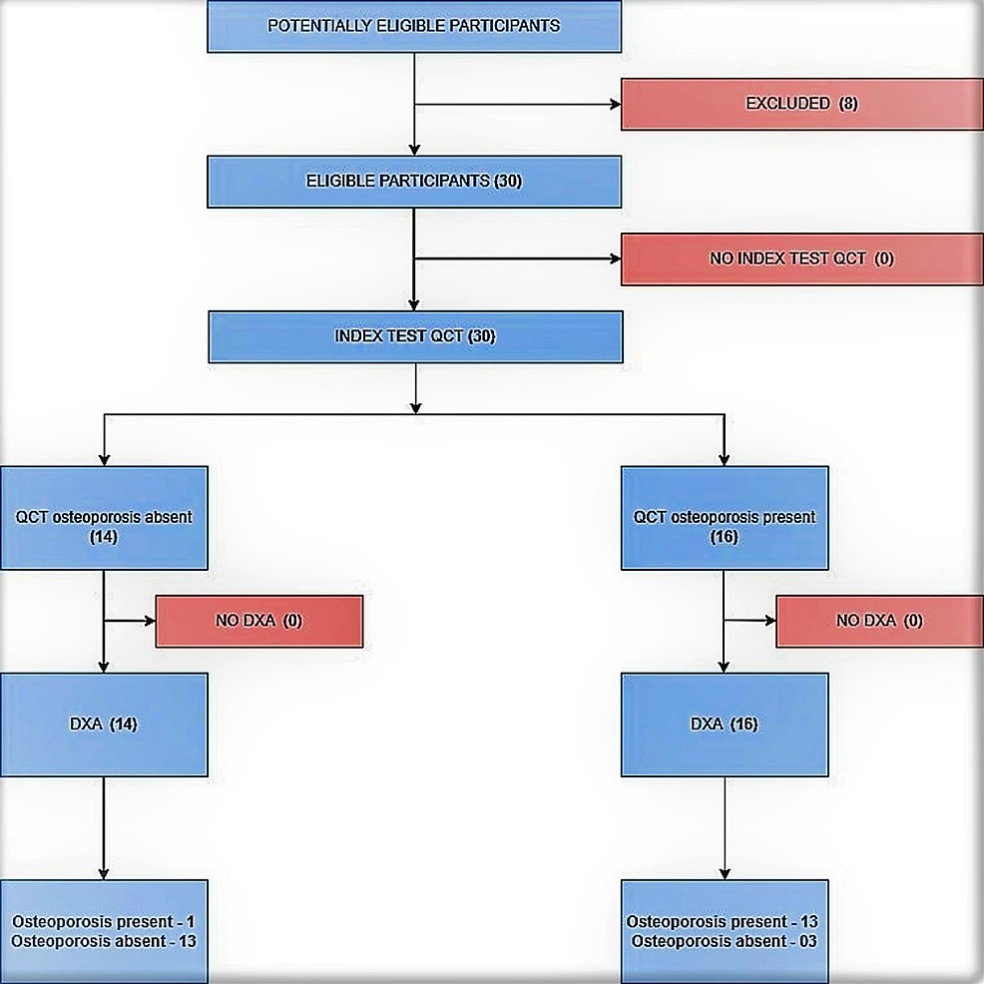
Study flow chart QCT: quantitative computed tomography; DXA: dual x-ray absorptiometry

A cross-sectional diagnostic accuracy study was conducted as per the Standards for Reporting Diagnostic Accuracy (STARD) 2015 guideline [9]. It was a pilot study on 30 participants [10]. The study flow chart is illustrated in Figure 1.

Operational definition

The ADA criteria for the diagnosis of diabetes were used [6]. The diagnostic criteria defined by the World Health Organization (WHO) in 1994 were used for DXA to diagnose osteoporosis. A T-score of <-2.5 standard deviation (SD) is diagnosed as osteoporosis, -1.0 to -2.5 SD suggests osteopenia, and >-1 SD is considered normal [11]. The criteria suggested by the American College of Radiology and the International Society for Clinical Densitometry were used for QCT [3,12]. For the spinal trabecular BMD measured by QCT, the thresholds are <120 mg/cm^3^ for osteopenia (corresponding to a DXA T-score of -1.0 SD) and <80 mg/cm^3^ for osteoporosis (corresponding to a DXA T-score of -2.5 SD). According to Woodson's definition, discrepancies in the diagnosis of osteoporosis between DXA and QCT were divided into two groups: minor and major [13]. Minor discordance was defined as when the difference between the two tools is no more than one WHO diagnostic class. Major discordance was present when one tool suggested osteoporosis and the other suggested normal BMD.

Data entry and analysis

Data were coded and recorded in the MS Excel spreadsheet program. IBM SPSS Statistics v26 (IBM Corp., Armonk, NY) was used for data analysis. Pearson's chi-square was used to calculate the p-value. Diagnostic discordance was done according to Woodson's definition [13]. The diagnostic accuracy was measured by sensitivity, specificity, positive predictive value (PPV), negative predictive value (NPV), positive and negative likelihood ratios, and the positive and negative post-test probabilities. A receiver operating characteristic (ROC) curve was drawn. Taking the confidence level as 95%, a p-value <0.05 was considered statistically significant.

Ethical considerations and confidentiality of data

The study was conducted after getting approval from the Institute Ethics Committee, AIIMS Rishikesh (ref. no. AIIMS/IEC/21/260; May 15, 2021). No additional charges were taken from the participants for the investigations. Confidentiality of the information about the patients was maintained and the identity of patients was not revealed. Data, if shared, were anonymized.

## Results

The current study showed that QCT, compared to the gold standard DXA for osteoporosis detection among patients with diabetes, has a sensitivity of 92.8% (95% CI 92.4%-93.2%) and specificity of 81.2% (95% CI 80.6%-81.8%) as depicted in Table 1. The PPV of QCT was 81.2% (95% CI 80.6%-81.8%) and NPV was 92.8% (95% CI 92.4%-93.2%).

**Table 1:** Sensitivity, specificity, PPV, NPV, likelihood ratio, and post-test probability of QCT PPV: positive predictive value; NPV: negative predictive value; QCT: quantitative computed tomography; CI: confidence interval

Result	Point estimate	Lower CI	Upper CI
Sensitivity	92.8%	92.4%	93.2%
Specificity	81.2%	80.6%	81.8%
PPV	81.2%	80.6%	81.8%
NPV	92.8%	92.4%	93.2%
Pre-test probability	46.6%		
Positive likelihood ratio	4.95	4.79	5.11
Positive post-test probability	0.81		
Negative likelihood ratio	0.087	0.082	0.093
Negative post-test probability	0.071		

**Figure 2:**
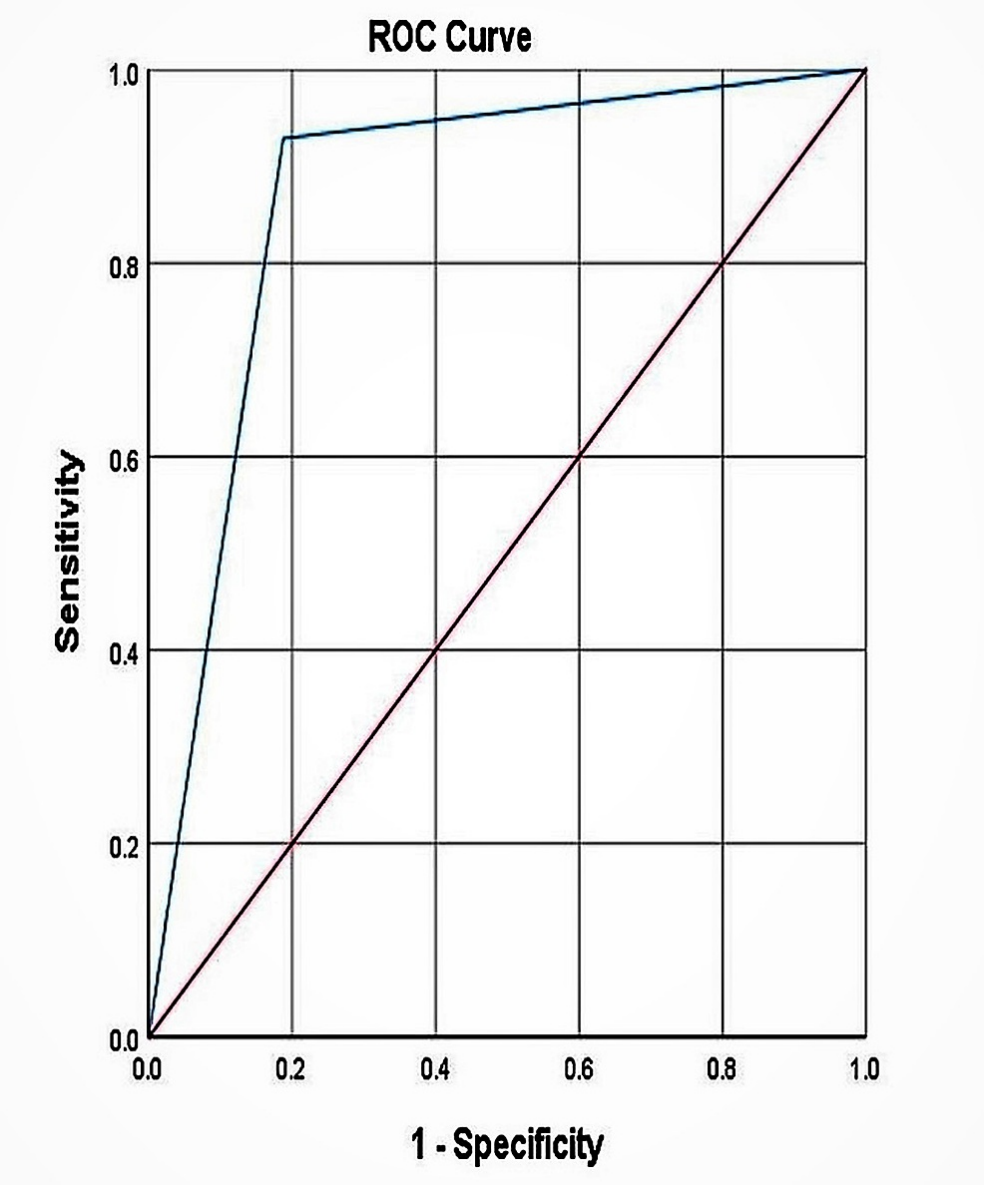
ROC curve of QCT when compared with the gold standard DXA in osteoporosis detection among diabetic patients ROC curve: receiver operating characteristic curve; QCT: quantitative computed tomography; DXA: dual x-ray absorptiometry

The pre-test probability for osteoporosis was 46.6% among individuals with diabetes. The positive likelihood ratio was 4.95 (95% CI 4.79-5.11), and the positive post-test probability was 0.81. The negative likelihood ratio was 0.087 (95% CI 0.082-0.093), and the negative post-test probability was 0.071 as depicted in Table 1. An ROC curve as shown in Figure 2 was plotted to compare the diagnostic performance of QCT, which revealed an area under the curve (AUC) of 0.871 (95% CI 0.731-1.00) when the osteoporosis cut-off was taken as <80 mg/cm^3^ as per international guidelines as shown in Table 2.

**Table 2:** Area under the curve of the ROC curve of QCT when compared with the gold standard DXA in osteoporosis detection among diabetic patients ROC curve: receiver operating characteristic curve; QCT: quantitative computed tomography; DXA: dual x-ray absorptiometry

Area under the curve				
Area	Std. error	Asymptotic sig.	Asymptotic 95% confidence interval	
			Lower bound	Upper bound
0.871	.071	.001	0.731	1.000

This study showed a diagnostic discordance in the diagnosis of osteoporosis between DXA and QCT in 36.6% of patients with diabetes, while no evidence of major discordance was present as shown in Table 3. The concordance was observed in 63.3% of cases. The current study showed that lumbar spine QCT detected osteoporosis in 53.3% of patients with diabetes, while DXA lumbar spine detected osteoporosis in 43.3% of patients with diabetes, and DXA hip detected osteoporosis in 36.6% of patients with diabetes. These discordances were minor discordance, presenting a difference of only one class.

**Table 3:** Distribution of diagnostic discordances between QCT and DXA QCT: quantitative computed tomography; DXA: dual x-ray absorptiometry

Diagnostic discordance, N (%)		n
Major discordance	QCT osteoporosis; DXA normal	0
	QCT normal; DXA osteoporosis	0
Minor discordance: 11 (36.6)	QCT osteoporosis; DXA osteopenia	3
	QCT osteopenia; DXA osteoporosis	1
	QCT osteopenia; DXA normal	7
	QCT normal; DXA osteopenia	0
Concordance: 19 (63.3)	QCT and DXA osteoporosis	13
	QCT and DXA osteopenia	5
	QCT and DXA normal	1

## Discussion

Albright and Reifenstein in 1948 described an association between diabetes and reduced bone mass, proving that DM might affect the skeletal system and result in osteoporosis. Following this, much attention has been paid to the association between diabetes and osteoporosis [14]. DM adversely affects the skeleton and is associated with an increased risk of osteoporosis and fragility fractures [15]. A systematic review that included 836,941 participants revealed a strong association between T1DM and T2DM and increased risk of hip fracture; also, the association between type of diabetes and hip fracture was stronger for T1DM (RR 6.3) than for T2DM (RR 1.7) [16]. These findings strongly endorse an association between T1DM and T2DM and increased risk of hip fracture in men and women.

Osteoporosis is a systemic skeletal disorder manifested as low-BMD, micro-architectural deterioration of bone tissue leading to bone fragility, and subsequent increase in fracture risk. In 1994, WHO gave an operational definition of osteoporosis based on BMD. The sites most frequently measured are the lumbar spine and hip. DXA and QCT are the most frequently used clinical techniques for BMD measurement. BMD measured by DXA is currently the gold standard for both osteoporosis diagnosis and the monitoring of treatment efficacy.

One significant distinction in technologies relates to monitoring. Spine BMD values measured by QCT demonstrate relatively increased rates of bone loss with advanced age compared with DXA values because of the exclusively cancellous bone measurements of QCT; the rate of change in cancellous bone is higher than that of cortical bone. QCT has been recognized for diagnosing osteoporosis by the American College of Radiology and the International Society for Clinical Densitometry [17]. In addition, QCT provides exclusively cancellous bone measurements, which are more susceptible to changes with disease affecting BMD and therapies for osteoporosis. Black et al. conducted a randomized double-blinded clinical study of antiresorptive drugs parathyroid hormone and alendronate to test the hypothesis that the concurrent administration of the two agents would increase bone density more than the use of either one alone; the study demonstrated that changes in BMD shown with QCT in patients treated with parathyroid drugs and alendronate were two to three times higher than those found with DXA [18].

Volumetric trabecular BMD measurement can have several advantages over DXA measurements. Since trabecular bone is affected earlier and to a greater extent than cortical bone, QCT is likely to detect low bone mass earlier in the spine [19]. Also, artificially high BMD measurements by DXA due to obesity, aortic calcification, osteophytes, and disc space narrowing, or degenerative spinal diseases can be avoided [20-23].

Comparisons of DXA and QCT applications have been the subject of several research studies. Among 140 postmenopausal women, Li et al. conducted a comparison of QCT and DXA and found that the detection rate was 17.1% for DXA and 46.4% for QCT (p<0.01); the study also demonstrated that QCT had a considerably greater detection rate of osteoporosis in postmenopausal women than DXA [4]. Similarly, Xu et al., in their study in 313 Chinese elderly men, showed that the osteoporosis detection rate was 10.9% for DXA and 45.1% for QCT (p<0.001) and demonstrated that QCT is a much more sensitive method for measuring BMD [5]. Mao et al. concluded that thoracic and lumbar QCT provides a similar and more sensitive method for detecting bone mineral loss when compared to DXA [24]. However, no such studies have been conducted to compare the diagnostic accuracy of QCT in patients with diabetes. The current study showed that QCT, compared to the gold standard DXA for osteoporosis detection among patients with diabetes, had a sensitivity of 92.8% and specificity of 81.2%. The PPV of QCT was 81.2% and NPV was 92.8%. When comparing lumbar spine QCT with DXA, a previous study among participants with no known comorbidities showed a sensitivity of 81.3, specificity of 93.3%, PPV of 92%, and NPV of 83% [25].

This study demonstrated a discordance in the diagnosis of osteoporosis between DXA and QCT in 36.6% of patients with diabetes, while no evidence of major discordance was present. The concordance was observed in 63.3% of cases. The current study showed that lumbar spine QCT detected osteoporosis in 53.3% of patients with diabetes, while lumbar spine DXA detected osteoporosis in 43.3% of patients with diabetes and hip DXA detected osteoporosis in 36.6% of patients with diabetes. These discordances were minor, presenting a difference of only one class. Xu et al., in their study, have shown that concordance, minor discordance, and major discordance between QCT and DXA were present in 40.9%, 50.8%, and 8.30% cases, respectively. The possible causes of these discordances were spine degeneration, vertebral fractures, and abdominal aorta calcification [5]. These discordances may have an impact on a patient's treatment approach and overall prognosis.

Greenspan et al. showed that vertebral fractures were present in 18.3% of asymptomatic postmenopausal women and that 11.0% to 18.7% of individuals with clinical osteoporosis would have been classified as having normal BMD by using DXA bone density criteria alone [26]. Our study found the history of the previous fracture in adult life occurring spontaneously, or a fracture arising from trauma which, in a healthy individual, would not have resulted in a fracture were present in three cases. In all these cases, QCT showed osteoporosis while DXA was suggestive of osteopenia. This study showed that DXA may have underestimated the extent of osteoporosis in these patients. According to the current study, utilizing DXA alone to diagnose osteoporosis may result in the condition being ignored, and DXA is insufficient to account for the entire spectrum of fracture risks in diabetic individuals.

Considering the silent nature of osteoporosis, and the prevalence in T2DM patients to be around 37.8%, a proactive approach is required to diagnose and treat osteoporosis [27]. The use of technology in the diagnosis of osteoporosis should be encouraged as tools like the FRAX have limited applicability in DM as the parameters related to DM are not included in FRAX. Schwartz et al. demonstrated that fracture risk was higher among older adults with T2DM than those without diabetes, even with a similar FRAX score [28].

To the best of our knowledge, this study is the first of its kind to assess the diagnostic accuracy of QCT in osteoporosis detection in patients with diabetes. Our study showed that QCT has a sensitivity of 92.8% (95% CI 92.4%-93.2%) and specificity of 81.2% (95% CI 80.6%-81.8%). The ROC curve revealed an AUC of 0.871 (95% CI 0.731-1.00) when the osteoporosis cut-off was taken as <80 mg/cm^3^. AUC is a powerful way to epitomize the overall diagnostic accuracy of the test. An AUC of 0.5 indicates no discrimination (i.e., the capacity to diagnose patients with and without the disease or condition based on the test), 0.7-0.8 is considered acceptable, 0.8-0.9 is considered excellent, and more than 0.9 is considered outstanding [29]. Another advantage with QCT is the ease of availability as QCT can be performed on most CT scanners. In contrast, Curtis et al. showed limited access of DXA, especially in a rural population, in a study of the geographic availability and associated utilization of DXA testing among older persons in the United States [30]. QCT is an alternative tool that is simple, quick, non-invasive, has excellent diagnostic accuracy, and is easily available. It has an important role in the evaluation of individuals at risk of osteoporosis, and in aiding clinicians in treating osteoporosis.

Based on these results, this study suggests that QCT may serve as a problem-solving investigation tool where DXA is unavailable, or it may be the primary investigation tool for BMD measurement and osteoporosis detection if a dedicated DXA scanner is inaccessible. This study demonstrated frequent discordance when using QCT and DXA to diagnose osteoporosis among individuals with diabetes. QCT can be a better modality to look for changes in the quality of bone, even before these changes are noticed by a DXA scan. While recognizing DXA as a reference standard, the current study suggests that the QCT scan may have some advantages over the DXA in the case of screening. The false interpenetration of BMD in DXA due to osteophytes and degenerative changes may overestimate the T-score result, which is avoided in CT. Additionally, picture archiving and communication system files can be used to compare the attenuation over time, adding value to the analysis of how the BMD changes over time, especially within the first two years after diagnosis or the beginning of treatment.

At the level of clinical practice, BMD assessment based on a CT scan provides a valuable benefit that should be utilized to overcome the silent and asymptomatic nature of the disease. This study recommends recognizing osteoporosis screening with every CT scan done for the abdominal area or the use of L1 vertebra as a routine screening since it is included on all standard chest and abdominal CT scans. This study recommends further investigating the feasibility of opportunistic osteoporosis screening in routine abdominal and chest CT.

This study also has some limitations. We did not conduct statistical analysis of all the potential risk factors of diagnostic discordance in patients with diabetes. Moreover, further prognostic studies with extended follow-up designs are needed to determine the impact of existing discordance on patients' prognosis and fracture risk. The study sample size was relatively small and cannot be generalized unless a further study with a higher sample size is conducted. Although the ionizing radiation dose of spinal QCT is higher than that for DXA, the dose compares favorably with doses of other radiographic procedures (spinal radiographs) performed in patients suspected of having osteoporosis; this study did not measure radiation exposure [19].

## Conclusions

DXA is the gold standard diagnostic tool; however, its availability is limited. The current study assessed the diagnostic accuracy of QCT in osteoporosis detection in people with diabetes and showed that QCT is an excellent diagnostic tool. Based on these results, this study recommends that QCT may serve as a problem-solving investigation tool where DXA is unavailable, or it may be the primary investigation tool for BMD measurement and osteoporosis detection if a dedicated DXA scanner is inaccessible. This study also recommends further investigating the feasibility of opportunistic osteoporosis screening in routine abdominal and chest CT. Finally, considering the silent nature of osteoporosis and the high prevalence of osteoporosis in individuals with diabetes, a proactive approach is required in the screening of osteoporosis.

## References

[1] Vestergaard P (2007). Discrepancies in bone mineral density and fracture risk in patients with type 1 and type 2 diabetes—a meta-analysis. Osteoporos Int.

[2] Zeitoun D, Caliaperoumal G, Bensidhoum M, Constans JM, Anagnostou F, Bousson V (2019). Microcomputed tomography of the femur of diabetic rats: alterations of trabecular and cortical bone microarchitecture and vasculature—a feasibility study. Eur Radiol Exp.

[3] Ward RJ, Roberts CC, Bencardino JT (2017). ACR Appropriateness Criteria® Osteoporosis and Bone Mineral Density. J Am Coll Radiol.

[4] Li N, Li XM, Xu L, Sun WJ, Cheng XG, Tian W (2013). Comparison of QCT and DXA: osteoporosis detection rates in postmenopausal women. Int J Endocrinol.

[5] Xu XM, Li N, Li K, Li XY, Zhang P, Xuan YJ, Cheng XG (2019). Discordance in diagnosis of osteoporosis by quantitative computed tomography and dual-energy X-ray absorptiometry in Chinese elderly men. J Orthop Translat.

[6] American Diabetes Association (2021). 2. Classification and Diagnosis of Diabetes: Standards of Medical Care in Diabetes—2021. Diabetes Care.

[7] Kanis JA, Hans D, Cooper C (2011). Interpretation and use of FRAX in clinical practice. Osteoporos Int.

[8] Garg MK, Kharb S (2013). Dual energy X-ray absorptiometry: pitfalls in measurement and interpretation of bone mineral density. Indian J Endocrinol Metab.

[9] Bossuyt PM, Reitsma JB, Bruns DE (2015). STARD 2015: an updated list of essential items for reporting diagnostic accuracy studies. BMJ.

[10] Kwak SG, Kim JH (2017). Central limit theorem: the cornerstone of modern statistics. Korean J Anesthesiol.

[11] Kanis JA, Kanis JA (1994). Assessment of fracture risk and its application to screening for postmenopausal osteoporosis: synopsis of a WHO report. Osteoporos Int.

[12] Engelke K, Adams JE, Armbrecht G (2008). Clinical use of quantitative computed tomography and peripheral quantitative computed tomography in the management of osteoporosis in adults: the 2007 ISCD Official Positions. J Clin Densitom.

[13] Woodson G (2000). Dual X-ray absorptiometry T-score concordance and discordance between the hip and spine measurement sites. J Clin Densitom.

[14] Dhaon P, Shah VN (2014). Type 1 diabetes and osteoporosis: a review of literature. Indian J Endocrinol Metab.

[15] Kumari C, Yagoub G, Ashfaque M, Jawed S, Hamid P (2021). Consequences of diabetes mellitus in bone health: traditional review. Cureus.

[16] Janghorbani M, Van Dam RM, Willett WC, Hu FB (2007). Systematic review of type 1 and type 2 diabetes mellitus and risk of fracture. Am J Epidemiol.

[17] Li X, Na L, Xiaoguang C (2014). Update on the clinical application of quantitative computed tomography (QCT) in osteoporosis. Curr Radiol Rep.

[18] Black DM, Greenspan SL, Ensrud KE (2003). The effects of parathyroid hormone and alendronate alone or in combination in postmenopausal osteoporosis. N Engl J Med.

[19] Adams JE (2009). Quantitative computed tomography. Eur J Radiol.

[20] Yu EW, Thomas BJ, Brown JK, Finkelstein JS (2012). Simulated increases in body fat and errors in bone mineral density measurements by DXA and QCT. J Bone Miner Res.

[21] Smith JA, Vento JA, Spencer RP, Tendler BE (1999). Aortic calcification contributing to bone densitometry measurement. J Clin Densitom.

[22] Reid IR, Evans MC, Ames R, Wattie DJ (1991). The influence of osteophytes and aortic calcification on spinal mineral density in postmenopausal women. J Clin Endocrinol Metab.

[23] Guglielmi G, Floriani I, Torri V, Li J, van Kuijk C, Genant HK, Lang TF (2005). Effect of spinal degenerative changes on volumetric bone mineral density of the central skeleton as measured by quantitative computed tomography. Acta Radiol.

[24] Mao SS, Li D, Syed YS (2017). Thoracic quantitative computed tomography (QCT) can sensitively monitor bone mineral metabolism: comparison of thoracic QCT vs lumbar QCT and dual-energy X-ray absorptiometry in detection of age-relative change in bone mineral density. Acad Radiol.

[25] Hassan NE, El-Masry SA, El-Banna RA, El Hussieny MS (2014). Different tools for the assessment of bone mass among Egyptian adults. Open Access Maced J Med Sci.

[26] Greenspan SL, von Stetten E, Emond SK, Jones L, Parker RA (2001). Instant vertebral assessment: a noninvasive dual X-ray absorptiometry technique to avoid misclassification and clinical mismanagement of osteoporosis. J Clin Densitom.

[27] SI Y, WA C, GU Y, XU G, MA Y (2019). Prevalence of osteoporosis in patients with type 2 diabetes mellitus in the Chinese Mainland: a systematic review and meta-analysis. Iran J Public Health.

[28] Schwartz AV, Vittinghoff E, Bauer DC (2011). Association of BMD and FRAX score with risk of fracture in older adults with type 2 diabetes. JAMA.

[29] Mandrekar JN (2010). Receiver operating characteristic curve in diagnostic test assessment. J Thorac Oncol.

[30] Curtis JR, Laster A, Becker DJ (2009). The geographic availability and associated utilization of dual-energy X-ray absorptiometry (DXA) testing among older persons in the United States. Osteoporos Int.

